# The DA VINCI study: is Ireland achieving ESC/EAS guideline–directed LDL-C goals?

**DOI:** 10.1007/s11845-022-03050-6

**Published:** 2022-07-01

**Authors:** Gregory Offiah, Cormac O’Connor, Cormac Kennedy, Joe Gallagher, Patricia O’Connor, Brendan McAdam, Kausik K. Ray, Marieke Schoonen, Vincent Maher

**Affiliations:** 1 Department of Cardiology, Tallaght University Hospital, Dublin, Ireland; 2 Lipid Clinic, St James Hospital, Dublin, Ireland; 3 The Palms GP Surgery, The Avenue, Gorey, Co. Wexford Ireland; 4 Department of Cardiology, Beaumont Hospital, Dublin, Ireland; 5 Royal College of Surgeons, Dublin, Ireland; 6 Imperial Centre for Cardiovascular Disease Prevention and Imperial Clinical Trials Unit, Imperial College London, London, UK; 7 Center for Observational Research, Amgen Ltd, Uxbridge, UK; 8 Advanced Lipid Management and Research ALMAR Centre, Tallaght University Hospital, Dublin, Ireland

**Keywords:** Guidelines, Lipids, Low-density lipoprotein cholesterol, Statins

## Abstract

**Background:**

The EU-wide, cross-sectional observational study of lipid-lowering therapy (LLT) use in secondary and primary care (DA VINCI) assessed the proportion of patients achieving low-density lipoprotein cholesterol (LDL-C) goals recommended by the European Society of Cardiology (ESC)/European Atherosclerosis Society (EAS) guidelines and provided an insight into regional use of LLT in Europe, including Ireland.

**Aims:**

This analysis focuses on data from patients in Ireland who participated in the DA VINCI study.

**Methods:**

The DA VINCI study enrolled patients receiving LLT at primary and secondary care sites across 18 European countries between June 2017 and November 2018. The study assessed the achievement of risk-based 2016 and 2019 ESC/EAS LDL-C goals. This subgroup analysis aimed to evaluate LDL-C goal attainment in an Irish cohort of primary and secondary care patients.

**Results:**

In total, 198 patients from Ireland were enrolled from three primary care and three secondary care centres. Most patients were White and male, and were receiving moderate- or high-intensity statin therapy (most frequently atorvastatin or rosuvastatin). Few patients (< 10%) were receiving combination therapy of statin and ezetimibe. Approximately 60% of patients achieved their 2016 ESC/EAC LDL-C goals while less than half the patients achieved their 2019 ESC/EAS goals. Approximately half of secondary prevention patients achieved their 2016 ESC/EAS goals and only 20% of secondary prevention patients achieved their 2019 ESC/EAS goals.

**Conclusions:**

These results highlight the disparity between dyslipidaemia management in clinical practice in Ireland and guideline recommendations.

**Trial registration:**

ENCePP; EU PAS 22,075; date registered 06 February 2018.

## Introduction

Cardiovascular (CV) disease (CVD) is a major cause of mortality and morbidity, accounting for 45% of all deaths in Europe [1]. In Ireland, an estimated 10,000 people die each year from CVD [2]. It is also associated with a burden on healthcare costs [3]. Overall, CVD-related healthcare costs, including hospitalisations and loss of productivity because of illness, are estimated to cost the EU economy €210 billion per year [1].

Elevated low-density lipoprotein cholesterol (LDL-C) level is a well-established causal risk factor for atherosclerotic cardiovascular disease (ASCVD) [4]. In addition, elevated LDL-C level is directly correlated with an increased risk of CV events, including myocardial infarction and ischaemic stroke [4, 5]. LDL-C reduction with lipid-lowering therapy (LLT) has been proven to lower the incidence of fatal and non-fatal CV events [5, 6]. CV primary and secondary prevention aims to improve patients’ health-related quality of life by reducing the risk of disease progression and hospitalisation. Several landmark trials investigating the efficacy of proprotein convertase subtilisin/kexin 9 inhibitors (PCSK9is) have demonstrated an additional mortality benefit associated with further reductions in LDL-C levels [7, 8]. This has been reflected in the European Society of Cardiology (ESC)/European Atherosclerosis Society (EAS) guidelines on dyslipidaemia management [9, 10]. The 2016 ESC/EAS guidelines recommended LDL-C goals of less than 2.6 mmol/L (100 mg/dL) for individuals at high risk and less than 1.8 mmol/L (70 mg/dL) for those at very high risk [9]. The updated 2019 ESC/EAS guidelines, based on high-quality clinical trial data, recommend more stringent LDL-C goals than those from 2016 for patients with established ASCVD and patients in the moderate-, high-, and very-high-risk primary prevention categories [10]. The updated 2019 ESC/EAS guidelines now recommend at least a 50% reduction in LDL-C levels from the untreated state in addition to LDL-C goals of less than 1.8 mmol/L (70 mg/dL) and less than 1.4 mmol/L (55 mg/dL) for those at high and very high risk, respectively [10]. Furthermore, a new, more aggressive goal of LDL-C less than 1.0 mmol/L has been established for individuals presenting with recurrent CV events [10].

Although it is well recognised that elevated LDL-C level is a causal risk factor for CV events, there remains a disparity between guideline-directed LDL-C goals and observational data on LDL-C levels achieved in real-world settings [4]. Several observational studies assessing the impact of the 2016 ESC/EAS dyslipidaemia guidelines, such as EUROASPIRE, revealed that a high percentage of patients failed to achieve their LDL-C goals [11, 12]. These findings have also been observed in more recent studies assessing the impact and attainment of the 2019 ESC/EAS dyslipidaemia guidelines [12, 13]. In these studies, the proportion of patients attaining their LDL-C goals was found to be even lower than that achieved with the 2016 goals [12, 13]. The EU-wide cross-sectional observational study of lipid-modifying therapy use in secondary and primary care (DA VINCI [ENCePP; EU PAS 22075]) set out to determine the proportion of patients across Europe achieving 2016 ESC/EAS risk-based LDL-C goals and provided an insight into how LLT was being employed in different regions [13]. One hundred and ninety-eight Irish patients were included in the original study. We performed a subgroup analysis of the Irish cohort to characterise the findings of this specific group and gain an understanding of whether CV disease is managed optimally in Ireland, as per guidelines.

## Methods

### Study design

The DA VINCI study was a cross-sectional observational investigation of 5888 adults receiving LLT in European clinical practice [13]. The study took place across 18 European countries between June 2017 and November 2018. Approximately equal numbers of primary and secondary prevention patients were recruited. Secondary prevention patients included those with coronary artery, cerebrovascular, or peripheral arterial disease. The study protocol was approved by all relevant ethics committees. Amgen sponsored both the original study and this subgroup analysis. The subgroup analysis of the Irish cohort of the DA VINCI study was performed with data collected from 198 patients as part of the original study.

### Study aims and outcomes

The DA VINCI study explored LDL-C goal attainment, based on individual CV risk, recommended by the 2016 and 2019 (post hoc analysis) ESC/EAS guidelines across Europe. The study aimed to provide an insight into variability in lipid management among diverse healthcare systems. LDL-C goal attainment was evaluated in patients at LDL-C measurement while receiving stabilised LLT, which was defined as no change in dose or regimen for at least 28 days before LDL-C measurement. The estimated 10-year CV risk at LDL-C measurement was established using the Systematic COronary Risk Evaluation (SCORE) for primary prevention patients and the REACH (REduction of Atherothrombosis for Continued Health) score for secondary prevention patients [14, 15].

Ireland was one of the countries included in the overall DA VINCI study. The current subgroup analysis aimed to assess LDL-C goal attainment in an Irish cohort of primary and secondary care patients.

### Data collection and analysis

Full eligibility criteria and methodology are available in the primary DA VINCI study report [13]. Briefly, patients were recruited at routine clinic visits. Only patients being prescribed LLT at enrolment or within 12 months before enrolment and having an LDL-C measurement recorded up to 14 months before enrolment were included. Data were collected using a standardised electronic case report form, and included baseline characteristics, background medical history, previous vascular history, LLT, and most recent lipid profile. Continuous variables were reported as mean, standard deviation, median, interquartile range, and minimum/maximum. Categorical data were reported as numbers and percentages.

## Results

### Patient characteristics, CV risk profile, and management

The DA VINCI study enrolled 5888 patients from 18 countries and 128 sites across Europe. The Irish cohort comprised 198 patients, from three primary care and three secondary care centres. The age of the patients enrolled ranged from 35 to 87 years, with a mean age of 64 years. Most patients were White and male. Similar baseline characteristics were observed in the Irish cohort and the overall European cohort (Table 1), but there tended to be more White men in the Irish study population (68%) as compared to the overall study (58%).

**Table 1:** Baseline patient characteristics and cardiovascular risk factors

Characteristic	Ireland *N* = 198	Europe *N* = 5888
Men	135 (68.2)	3413 (58.0)
Women	63 (31.8)	2475 (42.0)
White	183 (92.4)	5435 (92.3)
Age (years), mean (SD)	63.7 (11.4)	65.1 (11.9)
BMI (kg/m^2^), median (Q1, Q3)	29.1 (25.6, 32.1)	28.0 (25.1, 31.6)
Non-smoker	87 (43.9)	2854 (48.5)
Ex-smoker	81 (40.9)	2059 (35.0)
Smoker	30 (15.2)	957 (16.3)
Missing	0 (0.0)	18 (0.3)
Hypertension	118 (59.6)	4138 (70.3)
Diabetes mellitus	54 (27.3)	2293 (38.9)
Type 1	4 (2.0)	178 (3.0)
Type 2	50 (25.3)	2112 (35.9)
Missing	0 (0.0)	3 (< 0.1)
Chronic kidney disease	3 (1.5)	680 (11.5)
Rheumatoid arthritis	6 (3.0)	84 (1.4)
Atrial fibrillation	28 (14.1)	675 (11.5)
Left ventricular hypertrophy	4 (2.0)	573 (9.7)
Haemorrhagic stroke	7 (3.5)	101 (1.7)

Data are presented as *n* (%) unless stated otherwise

*BMI* body mass index, *Q* quartile, *SD* standard deviation

There was a similar proportion of non-smokers and ex-smokers in the Irish and European cohorts, with proportionally less active smokers in both groups (Table 1). Hypertension (59.6% and 70.3%, in the Irish and European cohorts, respectively) and diabetes mellitus (27.3% and 38.9%, in the Irish and European cohorts, respectively) were the two most prevalent medical comorbidities in both groups, with a tendency for a lower prevalence of hypertension, type 2 diabetes mellitus, chronic kidney disease, and left ventricular hypertrophy in the Irish cohort than in the European cohort (Table 1).

**Fig. 1:**
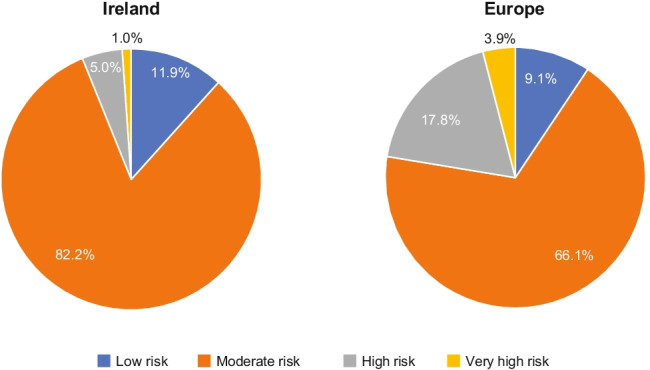
Primary prevention risk categories by Systematic COronary Risk Evaluation (SCORE)

A total of 101 Irish primary prevention patients participated in the study, of whom, 74 were evaluable for LDL-C goal attainment. The majority of primary prevention patients were in the moderate-risk category, measured by SCORE, in both the Irish and overall European cohorts (Fig. 1). Overall, 101 Irish secondary prevention patients were enrolled in the study, and 70 were evaluable for goal attainment. Table 2 describes the manifestations of established ASCVD documented for the patients, as well as previous vascular interventions. By definition, all of these patients were categorised as being at very high risk and were therefore assigned a more stringent LDL-C goal than low-, moderate-, or high-risk patients in both the 2016 and 2019 guidelines.

**Table 2:** Vascular history

	Ireland *N* = 198	Europe *N* = 5888
Family history of cardiovascular disease	52 (26.3)	969 (16.5)
Cardiovascular disease		
STEMI	6 (3.0)	324 (5.5)
NSTEMI	16 (8.1)	220 (3.7)
PCI	18 (9.1)	353 (6.0)
CABG	14 (7.1)	248 (4.2)
Stable angina	0 (0.0)	76 (1.3)
Unstable angina	2 (1.0)	38 (0.6)
Cerebrovascular disease		
Ischaemic stroke	31 (15.7)	961 (16.3)
Transient ischaemic attack	8 (4.0)	272 (4.6)
Carotid endarterectomy	7 (3.5)	94 (1.6)
Carotid artery stenting	2 (1.0)	47 (0.8)
Peripheral arterial disease		
Peripheral vascular stenting	2 (1.0)	382 (6.5)
Peripheral vascular bypass	1 (0.5)	211 (3.6)
Symptomatic claudication	17 (8.6)	355 (6.0)
Claudication	16 (8.1)	310 (5.3)
Other	5 (2.5)	252 (4.3)

Data are presented as *n* (%) unless stated otherwise

*CABG* coronary artery bypass grafting, *NSTEMI* non-ST-segment elevation myocardial infarction, *PCI* percutaneous coronary intervention, *STEMI* ST-segment elevation myocardial infarction

Most Irish patients were treated with statins (94.1%; most frequently atorvastatin or rosuvastatin). Compared to the overall study population, Irish patients more frequently received high-intensity statins (43.1% and 31.8%, respectively) (Table 3). However, less than half of the Irish patients had had their medication titrated onto the high-intensity statin doses. Overall, 8.6% of patients had a history of statin intolerance and 8.6% had reports of statin side effects. A minority of patients received combination therapy or non-statin LLT (Table 3). In the Irish cohort, 13 patients were receiving a statin/ezetimibe combination, one patient was receiving a statin/PCSK9i combination, with eight patients receiving non-statin LLT (bile acid sequestrants, fibrates, and fish oils).

**Table 3:** Stabilised lipid-lowering therapy at time of LDL-C measurement

Lipid-lowering therapy	Ireland *n* = 153^a^	Europe *n* = 4112^a^
Any^b^ statin	144 (94.1)	3856 (93.8)
Any^b^ high-intensity statin	66 (43.1)	1306 (31.8)
Any^b^ moderate-intensity statin	69 (45.1)	2279 (55.4)
Any^b^ low-intensity statin	9 (5.9)	171 (4.2)
High-intensity statin monotherapy	59 (38.6)	1134 (27.6)
Moderate-intensity statin monotherapy	63 (41.2)	2131 (51.8)
Low-intensity statin monotherapy	9 (5.9)	148 (3.6)
Any^b^ ezetimibe	20 (13.1)	491 (11.9)
Ezetimibe combination	13 (8.5)	380 (9.2)
PCSK9i combination	1 (0.7)	49 (1.1)
Other LLT^c^	8 (5.2)	270 (6.6)

Data are presented as *n* (%) unless stated otherwise

*PCSK9i* proprotein convertase subtilisin/kexin type 9 inhibitor

^a^Patients evaluable for LDL-C goal attainment

^b^Treatment given as monotherapy or in combination

^c^Bile acid sequestrants, fish oils, or fibrates

### ESC/EAS guideline LDL-C goal attainment

**Fig. 2:**
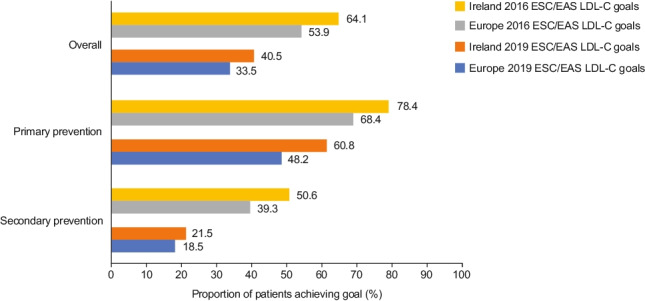
Attainment of LDL-C goals recommended by the 2016 and 2019 ESC/EAS guidelines. *EAS* European Atherosclerosis Society, *ESC* European Society of Cardiology, *LDL-C* low-density lipoprotein cholesterol

Forty-five Irish patients were excluded from the goal attainment analyses as they were not receiving stabilised LLT at the time of LDL-C measurement. Of the 153 Irish patients with evaluable data for LDL-C goal attainment, 64.1% (*n* = 98 [95% confidence interval (CI): 56.2–71.2]) of patients had achieved their LDL-C goals as per the 2016 ESC/EAS dyslipidaemia guidelines (Table 4, Fig. 2). In the post hoc analysis of data for the 2019 ESC/EAS guidelines, only 40.5% (*n* = 62 [95% CI: 33.1–48.4]) of Irish patients achieved their LDL-C goals (Table 4, Fig. 2).

**Table 4:** Low-density lipoprotein cholesterol goal attainment

	Ireland	Europe
Overall	*n* = 153	*n* = 4112
Achieved 2016 goal	98 (64.1)	2218 (53.9)
Achieved 2019 goal	62 (40.5)	1377 (33.5)
Primary prevention	*n* = 74	*n* = 2073
Achieved 2016 goal	58 (78.4)	1417 (68.4)
Achieved 2019 goal	45 (60.8)	1000 (48.2)
Secondary prevention	*n* = 79	*n* = 2039
Achieved 2016 goal	40 (50.6)	801 (39.3)
Achieved 2019 goal	17 (21.5)	377 (18.5)
Coronary artery disease	*n* = 20	*n* = 470
Achieved 2016 goal	10 (50)	207 (44.0)
Achieved 2019 goal	6 (30)	96 (20.4)
Cerebrovascular disease	*n* = 27	*n* = 751
Achieved 2016 goal	16 (59.3)	268 (35.7)
Achieved 2019 goal	5 (18.5)	122 (16.2)
Peripheral arterial disease	*n* = 32	*n* = 818
Achieved 2016 goal	14 (43.8)	326 (39.9)
Achieved 2019 goal	6 (18.8)	159 (19.4)

Data are presented as *n* (%) unless stated otherwise

**Fig. 3:**
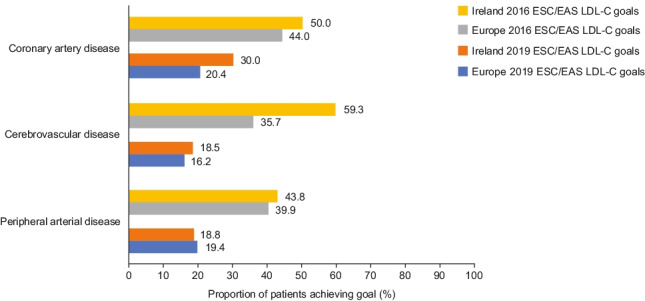
LDL-C goal attainment by vascular bed involvement. *EAS* European Atherosclerosis Society, *ESC* European Society of Cardiology, *LDL-C* low-density lipoprotein cholesterol

Achievement of LDL-C goals among the secondary prevention cohort was examined according to the patient’s main vascular bed involvement (coronary artery, cerebrovascular, or peripheral arterial disease). With the exception of patients with cerebrovascular disease, LDL-C goal attainment was low in all groups, with less than or half of the patients achieving their goals (Fig. 3).

According to additional analyses of the Irish cohort by gender and age, males were numerically more likely to achieve their LDL-C goals than females as per the 2016 ESC/EAS guidelines (67.9% versus 55.3%, respectively), but this trend was not seen for the attainment of the 2019 ESC/EAS guidelines (39.6% versus 42.6%). With respect to age, patients > 64 years were numerically more likely than those ≤ 64 years to achieve their LDL-C goals as per both the 2016 ESC/EAS guidelines (68.0% versus 60.3%, respectively) and the 2019 ESC/EAS guidelines (45.3% versus 35.9%).

## Discussion

This subgroup analysis set out to assess LDL-C goal attainment in the Irish cohort of the DA VINCI study. Over half of the patients in Ireland who participated in DA VINCI did not achieve the 2019 ESC/EAS LDL-C goals. Similar findings were identified across Europe in the DA VINCI study, as previously reported [13]. These results highlight the disparity between dyslipidaemia management in clinical practice in Ireland compared with guideline recommendations.

The 2016 guidelines were in effect at the time of data collection, yet only half of the patients reached their LDL-C goal. These findings are concerning, given that every patient in this cohort had proven vascular disease and many had already experienced a CV event or required vascular intervention. Despite the guidelines pertaining to all forms of established ASCVD, particular attention is paid to outcomes in coronary artery disease in the literature. Our study highlights poor adherence to guidelines for patients with cerebrovascular disease and peripheral arterial disease. When the more stringent 2019 ESC/EAS LDL-C guidelines were applied, as expected, an even smaller proportion of patients (Irish and overall) reached their LDL-C goal. In the Irish secondary prevention cohort, this was particularly low, with nearly four in five patients failing to reach their LDL-C goal. Among patients with established ASCVD, those with coronary artery disease had the highest rate of goal attainment, with lower rates observed in the cerebrovascular and peripheral arterial disease cohorts. More large-scale clinical trials are required to call to attention the benefits of strict LDL-C control in these cohorts.

As was seen in the secondary prevention subgroup, a lower proportion of patients in the primary prevention cohort achieved the more stringent 2019 risk-based LDL-C goals compared with the 2016 goals. However, compared with the secondary prevention cohort, a higher proportion of primary prevention patients met their LDL-C goals with statin monotherapy. In this regard, it should be noted that the majority of these primary prevention patients were in the moderate-risk category, whereas all of the secondary prevention patients were categorised as very high risk. The ESC/EAS LDL-C goals for high-/very-high-risk patients are considerably more stringent than those at moderate risk. Therefore, the difference in proportions of goal attainment between the primary and secondary prevention cohorts was likely influenced by the distribution of lower patient risk in the primary prevention category (i.e. it is easier to attain LDL-C goals when at lower risk because the levels are not as stringent).

The findings described here are in line with those of previous studies of Irish patients with dyslipidaemia [16]. Irish data from the Dyslipidemia International Study (DYSIS) demonstrated that a significant number of patients had persistent dyslipidaemia despite statin therapy and that patients in Ireland failed to reach their LDL-C goals [16]. Our data from the Irish cohort in the DA VINCI study were also consistent with the results from previous observational studies examining LDL-C goal attainment among patients with ASCVD in Europe [17]. In EUROASPIRE, a large cross-sectional study of patients in Europe, most patients with coronary heart disease did not achieve their 2016 LDL-C goals [17]. Among patients on high-intensity statin therapy, only 27% achieved LDL-C < 1.8 mmol/L. Similarly, a study from the START registry in Italy examining goal attainment using the 2016 and 2019 ESC/EAS guidelines showed that 58% of patients achieved their 2016 LDL-C goals, while only 3% achieved their 2019 LDL-C goals [12]. As was also observed in our analysis, LDL-C goal attainment reduced considerably when 2019 goals were applied [10]. Findings from EUROASPIRE suggested that men and those at least 60 years of age may have been somewhat more likely to achieve their goals [17], and we observed similar trends in our analysis.

Given the hypothesis that ‘lower is better’, it is essential that we identify and address inadequacies in dyslipidaemia management. The 2019 ESC/EAS guidelines suggest up-titration of statins and subsequent addition of non-statin LLT to achieve LDL-C goals, with expeditious follow-up for a reassessment of LDL-C levels [10]. Despite these recommendations and the observed low level of LDL-C goal attainment, less than half the patients in the Irish cohort were treated with high-intensity statins. Furthermore, only 20 patients received ezetimibe (13 in combination) and nine patients were treated with other forms of non-statin LLT (PCSK9is, bile acid sequestrants, fish oils, or fibrates). Compared with other European countries, limited resources are available for dyslipidaemia management in Ireland, with only six designated lipid clinics and one specialist lipid nurse being available [18] and no structured programme in primary care for primary prevention and a limited secondary prevention programme implemented to date. Thus, the development of a structured care programme in primary care and education on guideline-directed lipid management in primary care and general secondary care clinics is of the utmost importance. Emphasis should be placed on achieving LDL-C goals according to EAS/ESC 2019 guidelines. In addition, choosing high-intensity statins for high-risk patients and close monitoring of patients with repeated LDL-C measurements should be encouraged to facilitate dose up-titration and use of combination therapy to achieve LDL goals.

Studies have shown that very low levels of LDL-C are not associated with detrimental health effects or concerns [4, 19]. Every 1 mmol/L reduction in LDL-C is associated with an approximately 21% reduction in major vascular events per annum [20]. As newer guidelines continue to lower the desired level of LDL-C, this raises the question of whether statins alone can allow us to meet the required goals. In this regard, non-statin LLT can be effective when prescribed in combination with statins, particularly when higher doses of statins cannot be tolerated owing to side effects. The addition of ezetimibe to statins has been shown to reduce LDL-C levels up to an additional 24%, resulting in improved CV outcomes [19]. With the emergence of PCSK9is, which reduce LDL-C levels by 50–60%, these benefits continue due to additional reductions in LDL-C concentration [7, 8]. In this study, only one Irish patient was treated with a PCSK9i, most likely because DA VINCI was conducted prior to reimbursement of PCSK9is in Ireland. One PCSK9i, evolocumab, was approved for reimbursement in July 2019; however, strict national reimbursement criteria (based partly on cost-effectiveness and budget impact analyses) limit its widespread use [21]. To date, other new cholesterol-lowering drugs such as bempedoic acid and inclisiran are not yet available in Ireland. Increasing the availability of novel, potent, non-statin LLTs may facilitate successful dyslipidaemia guideline adherence in Ireland, thereby improving patient outcomes. Future analyses from the overall DA VINCI population will be conducted to better understand how optimising treatment—including optimisation of statin monotherapy, as well as combination therapy (in accordance with ESC/EAS dyslipidaemia guidelines)—impacts on the risk of subsequent cardiovascular events.

Our study had limitations. It was a cross-sectional observational study; as such, LDL-C levels were only measured at a single point in time. Importantly, there are no data available on medication compliance, drug intolerance, or changes in LDL-C levels following dose titration. The data were also collected before the publication of the 2019 ESC/EAS guidelines. Thus, post hoc analysis against newer goals was performed with the knowledge that patients were being treated using the more lenient 2016 ESC/EAS guidelines. If the study was to be repeated now, at a time when the lower LDL-C goals are more routinely used, we are hopeful that goal attainment would be higher when 2019 goals were applied.

## Conclusion

Our study demonstrates that dyslipidaemia management is suboptimal in Ireland, even though well-established guidelines (based on high-quality evidence) are available to healthcare professionals.

In secondary prevention patients, nearly half failed to achieve the 2016 LDL-C goals. Emphasis should be placed on prescriber education with respect to the following: current dyslipidaemia management guidelines, the correct use of statins, appropriate use of combination therapy, and appropriate patient monitoring. Patient education regarding medication adherence is also important. In high-risk patients with inadequate LDL-C lowering on maximally tolerated conventional therapy, including patients with familial hypercholesterolaemia, and those with established CV disease, consideration should be given to the use of additional non-statin LLT to achieve LDL-C goals.

## Data Availability

Qualified researchers may request data from Amgen clinical studies. Complete details are available at https://wwwext.amgen.com/science/clinical-trials/clinical-data-transparency-practices.
